# Effects of Sodium Selenate on Growth, Selenium Forms, and Nutritional Quality of *Chlorella pyrenoidosa*

**DOI:** 10.3390/foods14030405

**Published:** 2025-01-26

**Authors:** Xianwei Zhao, Jiali Jiang, Sushu Yang, Huimin Sun, Qingling Zhu, Yangyang Zhang, Zhuqing Zhao, Denghang Yu, Meiqin Zhuo

**Affiliations:** 1 Hubei Key Laboratory of Animal Nutrition and Feed Science, School of Animal Science and Nutritional Engineering, Wuhan Polytechnic University, Wuhan 430023, China; jiangjl200112@163.com (J.J.); yangsushu1998@163.com (S.Y.); sunhuimin020719@163.com (H.S.); yudenghang1985@163.com (D.Y.); 2 College of Resources and Environment, Huazhong Agricultural University, Wuhan 430070, China; zhaoxianwei980257@163.com (X.Z.); 130zhangyy@163.com (Y.Z.); zzq@mail.hzau.edu.cn (Z.Z.); 3 Marine Science and Technology College, Zhejiang Ocean University, Zhoushan 316000, China; zql1226@163.com

**Keywords:** *C. pyrenoidosa*, Na_2_SeO_4_, selenium forms, nutritional quality

## Abstract

In this study, *C. pyrenoidosa* were cultured with seven different concentrations of Na_2_SeO_4_ (0–10 mg/L), and the effects of Na_2_SeO_4_ on the growth, Se-forms, and nutritional quality of *C. pyrenoidosa* were explored. The results showed that at the concentration of 0.5 mg/L Na_2_SeO_4_, the *C. pyrenoidosa* were plump and healthy; the contents of biomass, soluble protein, lipids, and TPUFA reached the highest level; the total Se content in *C. pyrenoidosa* increased with the increasing Na_2_SeO_4_ concentrations. However, the proportion of organic Se in *C. pyrenoidosa*. reached the highest value of 87.58% at the concentration of 0.5 mg/L Na_2_SeO_4_. Among organic Se forms, SeMet accounted for the largest proportion, while MeSeCys accounted for a relatively smaller proportion, but SeCys_2_ was not detected. The addition of Na_2_SeO_4_ (except for ≤0.5 mg/L) reduced the contents of photosynthetic pigments in *C. pyrenoidosa*. In addition, the antioxidant capacity of *C. pyrenoidosa* first increased and then decreased with the increasing Na_2_SeO_4_ concentrations, but different enzymes exhibited different tolerances to Na_2_SeO_4_. Based on the above research results, 0.5 mg/L Na_2_SeO_4_ concentration is recommended for the production of Se-rich *C. pyrenoidosa*. Our findings will provide a theoretical basis and practical references for the development of Se-rich *C. pyrenoidosa* health care products.

## 1. Introduction

Selenium (Se) is an essential trace element for human life activities. It plays important biological functions, like antioxidant, immune regulation, thyroid hormone metabolism participation, and reproductive support [1,2,3]. Generally, humans mainly replenish Se by taking in organic Se. Selenomethionine (SeMet), selenocysteine (SeCys), and selenocystine (SeCys_2_), which are formed due to Se, substitute the sulfur in methionine, cysteine, and cystine, respectively [4]. They are not only incorporated into protein synthesis but also serve as a source of Se for the synthesis of Selenoproteins, which play important roles in antioxidant and redox biology (methionine-sulfoxide reductase (MsrB1), thioredoxin reductases (Txnr) and glutathione peroxidases (GSH-Px) and modulate ROS levels (Selenoprotein T, Selenoprotein S and Selenoprotein P) [5,6]. However, Se mainly exists in the inorganic form in nature, which is highly toxic with low digestion and absorption. Therefore, inorganic Se needs to be converted into organic Se by organisms [7]. In recent years, the microalgae represented by *C. pyrenoidosa* have become a hot topic of research on Se-rich foods because the *C. pyrenoidosa* are rich in protein, polyunsaturated fatty acids, and carotenoids, and they can efficiently absorb inorganic Se in the environment and convert it into organic Se [8,9,10].

In current studies, the most common method for algal Se enrichment is to add exogenous sodium selenite (Na_2_SeO_3_) or Na_2_SeO_4_ as a Se source into algal growth environments [11,12]. The absorption rate and tolerance rate of different algae to Na_2_SeO_3_ and Na_2_SeO_4_ vary greatly. For example, Guimarães et al. [13] have found that the absorption and utilization rate of Na_2_SeO_3_ is four times as much as that of Na_2_SeO_4_ via *Nannochloropsis oceanica*. In contrast, several studies also found that the absorption and utilization rates of Na_2_SeO_4_ were higher than those of Na_2_SeO_3_ via *Chlamydomonas reinhardtii* and *Chlorella vulgaris* [14,15,16]. Although many studies have reported the absorption and conversion mechanisms of Na_2_SeO_3_ via *C. pyrenoidosa* [8,9,10,17], few reports on the absorption rate and conversion efficiency of Na_2_SeO_4_ via *C. pyrenoidosa* are available. Zhao et al. [9] found that both total Se and organic Se content increased with the increasing Na_2_SeO_3_ concentration in *C. pyrenoidosa*, but the organic Se conversion rate showed a gradual decrease trend. The Hubei Provincial Health Commission stipulated in 2022 that the proportion of organic Se in safe Se-rich products must be more than 80% [18]. Previous studies have shown that organic Se in algal cells mainly has three forms, namely, SeMet, SeCys, and SeCys2 [13,19]. Therefore, when producing Se-rich products, the total Se content in the products, especially the form, content, and proportion of organic Se, should be taken into account. However, so far, the effect of Na_2_SeO_4_ on the *C. pyrenoidosa* has not been studied.

The growth status and nutritional quality of the algae are very important for the production of Se-rich foods. Previous studies showed that Se has a dual effect on the growth of algae. High concentrations of Se will inhibit algae growth, as well as reduce its’ antioxidant capacity by decreasing the activities of the superoxide dismutase (SOD) and catalase (CAT), and GSH-Px [8,9]. Excessive concentrations of Se can also reduce the nutritional quality of algae by reducing the synthesis of the protein, total unsaturated fatty acids (TUFAs), photosynthetic pigments, and others in algae [8,19]. In this study, for the first time, *C. pyrenoidosa* was used as a Se-rich carrier; Na_2_SeO_4_ was used as an exogenous Se; and the BG11 nutrient solution containing different concentrations of Na_2_SeO_4_ was employed as a medium to culture *C. pyrenoidosa* so as to explore the effects of different Na_2_SeO_4_ concentrations on the growth, health status, and nutritional quality of *C. pyrenoidosa*. Our findings will provide a theoretical basis for the development of nutritious and healthy Se-rich algal.

## 2. Materials and Methods

### 2.1. Experimental Materials and Reagents

*C. pyrenoidosa* (FACHB-9) and BG11 nutrient solutions were purchased from the Institute of Hydrobiology, Chinese Academy of Sciences (Wuhan, China). The Na_2_SeO_4_ was purchased from Shandong West Asia Chemical Co., Ltd. (Liaocheng, China). (MDA, BC0020), (SOD, BC0170), (POD, BC0090), (CAT, BC0200), and (GSH-Px, BC1170) detection kits were obtained from Solaibao Technology Co., Ltd. (Beijing, China).

### 2.2. Culture and Collection of C. pyrenoidosa

The nutrient solution with a glucose concentration of 5 g/L and a carbon–nitrogen ratio of 5:1 was prepared by adding glucose and sodium nitrate to the BG11 basic nutrient solution. The nutrient solution was poured into a 250 mL triangular flask and added with the Na_2_SeO_4_ solution to pre-prepare the final volume of the 100 mL of culture solution containing 0, 0.2, 0.5, 1, 3, 5, and 10 mg/L Na_2_SeO_4_, respectively. Each treatment has three replicates, and three independent experiments were carried out. The culture solution with the initial pH 6.0 was sterilized in a sterilizing pot at 121 °C for 20 min and inoculated with *C. pyrenoidosa* at the exponential growth phase (initial optical density OD_680_ = 0.1, dry weight ≈ 0.0195 g/L) on a sterile operating table. The *C. pyrenoidosa* was incubated at 28 °C in a horizontal constant-temperature shaker (180 r/min) with a light intensity of 8000 lux and a photo period of 24 h light: 0 h dark for 5 d. The *C. pyrenoidosa* were collected at the plateau stage, and its relevant indicators were detected.

### 2.3. Determination of C. pyrenoidosa Biomass and Growth Inhibition Rate

The biomass of *C. pyrenoidosa* was measured by dry weight method, as described by Zhao et al. [20]. The collected fresh *C. pyrenoidosa* were washed three times with ultrapure water. When the Se was undetectable in the supernatant by a hydride generation atomic fluorescence spectrometer (the method detection limit was 0.01 μg/L), the *C. pyrenoidosa* were freeze-dried, and the dry weight was weighed with an analytical balance. The growth inhibition rate of *C. pyrenoidosa* was calculated according to formula (1): growth inhibition rate (%) = (Nc − Nt)/Nc × 100%. In the formula, Nc represents the final biomass of the *C. pyrenoidosa* in the control group, and Nt indicates the final biomass of *C. pyrenoidosa* under Se-addition treatments.

### 2.4. Determination of Se Forms in C. pyrenoidosa

In total, 0.5 g of dry *C. pyrenoidosa* was microwave-digested with HNO_3_–HClO_4_ (4:1) mixed acid. The total Se content was measured using a hydride generation atomic fluorescence spectrometer via the previously reported method in our lab [21]. The contents of SeMet, MeSeCys, and SeCys_2_ in *C. pyrenoidosa* were determined by Stander Kechuang Pharmaceutical Technology Co., LTD. (Qingdao, China) using a liquid chromatograph-mass spectrometer. Briefly, the 0.5 g of dry *C. pyrenoidosa* were mixed with 5 mL of the pepsin XIV solution and subjected to ultrasonic treatment in a 37 °C water bath for 1 h. After extraction, the supernatant was obtained via centrifugation and filtered by a 0.45 μm filter membrane before analysis. The contents of MeSeCys, SeMet, and SeCys_2_ in *C. pyrenoidosa* were determined using standard curves prepared under the same conditions, with the known concentrations of MeSeCys, SeMet, and SeCys_2_, respectively. The volatile Se content was determined using the following equation, as reported in the study of Wang et al. [19]: *C_volatile_ = C_initial_ − C_medium_ − C_algae_*. *C_initial_* represent the initial concentration of the Se added, *C_medium_* represents the residue concentration of Se in the medium, and *C_algae_* represents the total Se content in *C. pyrenoidosa*.

### 2.5. Microscopic and Ultrastructural Observation of C. pyrenoidosa

The microstructure observation of *C. pyrenoidosa* under the concentrations of 0, 0.5, and 1 mg/L Na_2_SeO_4_ was performed by using the method previously reported with a minor modification [22]. Specifically, the *C. pyrenoidosa* was fixed with Lugol’s reagent for 5 min and observed under a microscope at ×400 times magnification, and the diameter of the *C. pyrenoidosa* was measured. The scanning electron microscopy (SEM) samples of *C. pyrenoidosa* were pre-processed, referring to Zheng et al. [23]. The apparent morphology of the processed *C. pyrenoidosa* sample was observed under a biological scanning electron microscope (JSM-6390LV) at ×8000 times magnification. The *C. pyrenoidosa* transmission electron microscopy (TEM) samples were pre-processed, referring to [20]. The processed *C. pyrenoidosa* TEM samples were observed with a 100 KV transmission electron microscope (H-7650) at ×15,000 times magnification.

### 2.6. Analysis of Nutritional Components of C. pyrenoidosa

The *C. pyrenoidosa* extract was obtained by using the method previously reported [24]. Briefly, 0.1 g of dry *C. pyrenoidosa* was mixed with 3 mL of the 3% potassium hydroxide solution, subjected to a 90 min boiling water bath, and *C. pyrenoidosa* were ultrasonicated for 9 min with an interval of 2 s between 4 s ultrasonication, and then *C. pyrenoidosa* extract was centrifuged. Subsequently, the soluble protein content was determined by using the Coomassie brilliant blue method. The content of total polysaccharides was determined by using the phenol-sulfuric acid method. The total lipid of *C. pyrenoidosa* was determined by using the method previously reported with slight modifications [25]. Specifically, 0.2 g of dry *C. pyrenoidosa* was resuspended in 3 mL of the mixed solution (chloroform: methanol: water volume ratio = 1:2:0.8), ultrasonically extracted for 15 min, then added with 1 mL of chloroform and 1 mL of distilled water, vortexed, stood for 1 min, and centrifuged at 6580× *g* for 5 min to collect the chloroform layer. The extraction process was repeated once. The chloroform layers from two extractions were combined and dried with nitrogen at 45 °C, and the oil content in the chloroform layers was determined by using the differential method. The fatty acid composition and component contents of *C. pyrenoidosa* were determined by Stander Kechuang Pharmaceutical Technology Co., Ltd. (Qingdao, China) using the gas chromatograph mass spectrometer. Briefly, the 0.2 g *C. pyrenoidosa* dry samples were subjected to hydrolysis, fat extraction, fat saponification, and fatty acid methyl esterification before measurements. The determination methods were used according to the previous reference described by Honicky et al. [26].

### 2.7. Determination of Photosynthetic Pigments

The photosynthetic pigments Chla, Chlb, and carotenoids in *C. pyrenoidosa* were measured according to previously reported method [27]. Specifically, 5 mL of the fresh *C. pyrenoidosa* samples were centrifuged for 5 min at 4110× *g*, and the supernatant was removed. Afterward, the *C. pyrenoidosa* were washed three times with distilled water and added with 5 mL methanol to extract photosynthetic pigments in the dark at 4 °C for 24 h. After further centrifugation, the absorbance at 665, 652, and 470 nm of Chla, Chlb, and the carotenoid in the supernatant was measured with a UV-visible spectrophotometer, respectively, and the concentration of each pigment (mg/L) was calculated according to the following formulae: Chla (mg/L) = 15.65 × A_665_ − 7.340 × A_652_; Chlb (mg/L) = 34.09 × A_652_ − 15.28 × A_665_; Carotenoid (mg/L) = (1000 × A_470_ − 1.633 × Chla − 104.9 × Chlb)/221.

### 2.8. Determination of MDA and Antioxidant Enzyme Activity

The *C. pyrenoidosa* were centrifuged at 4110× *g* for 10 min, and the supernatant was discarded. The precipitate was rinsed repeatedly with deionized water until the medium attached to the algae was completely removed. The *C. pyrenoidosa* precipitate was placed in a pre-cooled phosphate buffer solution (PBS) and ultrasonically disrupted on ice, and the homogenate was centrifuged at 8220× *g* for 30 min. The supernatant was collected for enzyme activity measurement. The total protein concentration in the supernatant was determined using the Coomassie brilliant blue method. The MDA content and enzyme activity of SOD, POD, CAT, and GSH-Px in *C. pyrenoidosa* were detected according to the kit’s instructions.

### 2.9. Data Analysis

One-way analysis of variance of the obtained data was performed using GraphPad Prism 9.5 statistical software. Each experiment was repeated three times. All the data were expressed as a mean ± standard deviation (SD) of three biological replicates (*n* = 3). *p* < 0.05 was considered as statistically significant.

## 3. Results

### 3.1. Effect of Na_2_SeO_4_ on Growth of C. pyrenoidosa

**Figure 1:**
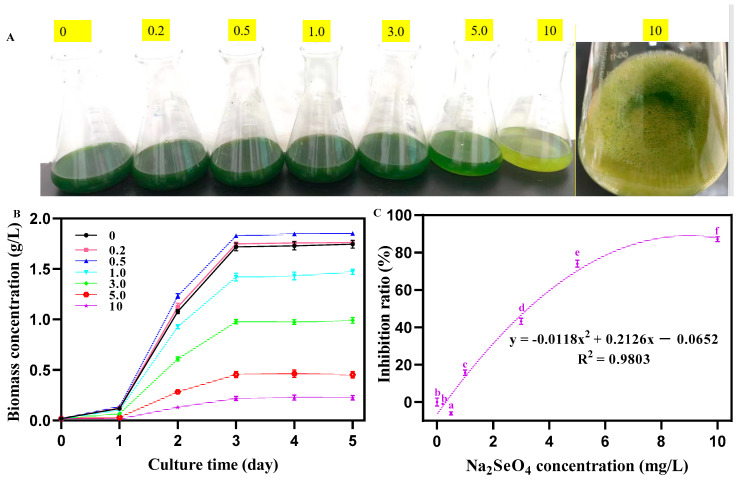
Growth status of *C. pyrenoidosa* under different concentrations of Na_2_SeO_4_. (**A**) Color of the algal liquid under different concentrations of Na_2_SeO_4_. (**B**) Biomass of *C. pyrenoidosa* under different concentrations of Na_2_SeO_4_. (**C**) Growth inhibition curve of *C. pyrenoidosa* under different concentrations of Na_2_SeO_4_. Data are expressed as mean ± SD (standard deviation, *n* = 3). Different lowercase letters represent significant differences. *p* ≤ 0.05 is considered statistically significant.

**Figure 2:**
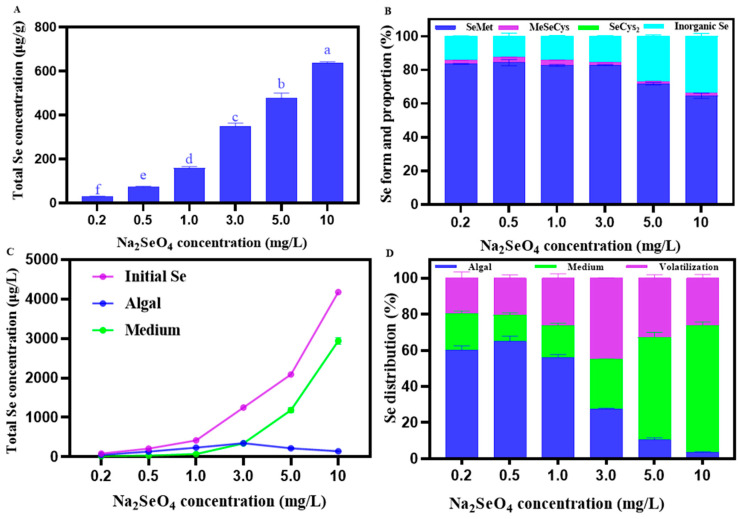
(**A**) Total Se content in *C. pyrenoidosa* under different Na_2_SeO_4_ addition concentrations. (**B**) Different Se forms and their proportions in *C. pyrenoidosa* under different Na_2_SeO_4_ addition concentrations. (**C**) Total Se content in the solution before Se absorption by *C. pyrenoidosa* (blue), the remaining solution after *C. pyrenoidosa* extraction (purple), *C. pyrenoidosa* (green). (**D**) Se distribution in the algae-water system under different Na_2_SeO_4_ addition concentrations. The volatile Se content was determined using the following equation: *C _volatile_ = C_initial_ − C_medium_ − C_algae_*. Data are expressed as mean ± SD (standard deviation) of three biological replicates. Different lowercase letters represent significant differences. *p* ≤ 0.05 is considered as significantly different.

With the increasing concentration of Na_2_SeO_4_, the color of the *C. pyrenoidosa* liquid gradually changed from dark green to light green. At a concentration of 10 mg/L Na_2_SeO_4_, algal liquid turned to light yellow, which was accompanied by the production of red substances (Figure 1A). As shown in Figure 1B,C, with the increasing concentration of Na_2_SeO_4_, the biomass of *C. pyrenoidosa* at 5 d first increased (0–0.5 mg/L) and then decreased (1–10 mg/L). The growth inhibition ratios of *C. pyrenoidosa* were −0.94%, −6.02%, 15.72%, 43.22%, 74.04%, and 84.10% under the concentrations of 0.2, 0.5, 1, 3, 5, and 10 mg/L Na2SeO4, respectively (Figure 2C). Theirs’ regression equation was obtained as follows:

y = −0.0118x^2^ + 0.2126x − 0.0652 (R^2^ = 0.9803)

### 3.2. Effect of Na_2_SeO_4_ Concentrations on Se Forms in C. pyrenoidosa

The total Se content in *C. pyrenoidosa* increased with the increasing Na_2_SeO_4_ concentrations, reaching the highest value of 638.36 ± 4.45 μg/g at the concentration of 10 mg/L Na_2_SeO_4_ (Figure 2A). As shown in Figure 2B, Se mainly existed in the form of organic Se in *C. pyrenoidosa*. With the increasing concentrations of Na_2_SeO_4_, the proportion of organic Se remained stable at (0–1.0 mg/L) and significantly decreased when the addition of Na_2_SeO4 > 3.0 mg/L. At a concentration of 0.5 mg/L Na_2_SeO_4_, the proportion of organic Se in *C. pyrenoidosa* reached the highest value of 87.58%, while at the concentration of 10 mg/L Na_2_SeO_4_, the lowest value of 66.11% was observed. Among organic Se forms, SeMet accounted for the largest proportion in Se, while MeSeCys accounted for a relatively smaller proportion, but SeCys_2_ was not detected. For example, at the concentration of 0.5 mg/L Na_2_SeO_4_, SeMet, and MeSeCys, respectively, accounted for 84.35 ± 1.81% and 3.22 ± 0.12% in the total Se of *C. pyrenoidosa*, with their contents being 61.84 ± 3.75 μg/g and 2.36 ± 0.18 μg/g, respectively. As shown in Figure 2C, at the concentration of 0–3.0 mg/L Na_2_SeO_4_, more Na_2_SeO_4_ was absorbed by the *C. pyrenoidosa*, relative to the Na_2_SeO_4_ remaining in the solution, and at the concentration of 3.0–10 mg/L Na_2_SeO_4_, the opposite trend was observed. As shown in Figure 2D, the percentage of Na_2_SeO_4_ absorbed by *C. pyrenoidosa* showed an initial increase and then a decrease with the increase in the applied Na_2_SeO_4_ concentration, reaching the highest value of 64.93 ± 2.31% and the lowest value of 3.44 ± 0.27% at 0.5 mg/L and 10 mg/L Na_2_SeO_4_ of the addition concentrations, respectively. Similarly, the percentage of volatile Na_2_SeO_4_ in *C. pyrenoidosa* showed a trend of first increasing and then decreasing with the increasing Na_2_SeO_4_ addition concentration, reaching the highest value of 45.03 ± 0.07% and the lowest value of 19.75 ± 2.71% at 3 mg/L and 0.2 mg/L Na_2_SeO_4_ concentrations, respectively. In contrast, the percentage of Na_2_SeO_4_ remaining in the solution first showed a decreasing and then an increasing trend, with the increasing Na_2_SeO_4_ concentration reaching the lowest value of 14.62 ± 0.99%, and the highest value of 70.32 ± 1.58% at the concentrations of the 0.5 mg/L and 10 mg/L Na_2_SeO_4_ concentrations, respectively.

### 3.3. Microscopic and Ultrastructural Observations of C. pyrenoidosa

**Figure 3:**
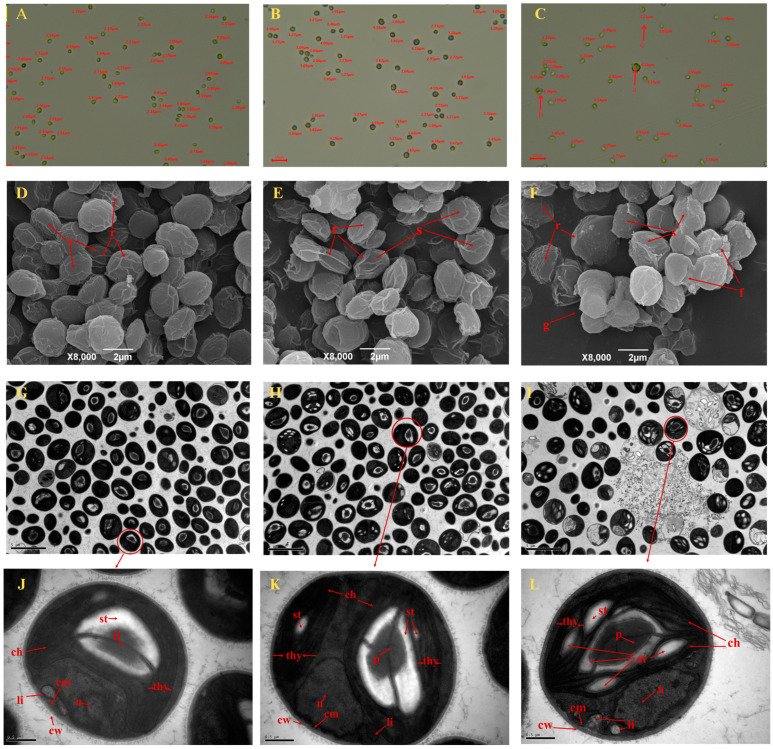
Representative diagram of microscopic and ultrastructural observations of *C. pyrenoidosa*. (**A**–**C**) Microscopic observation of the diameter and structure of *C. pyrenoidosa* at different concentrations of Na_2_SeO_4_ under the optical microscope: a, splitting; b, agglomeration; c, cavity phenomenon. (**D**–**F**) Scanning electron microscopy photos of *C. pyrenoidosa* at concentrations of 0, 0.5, and 1.0 mg/L Na_2_SeO_4_, respectively. s, cell surface; r, ridge of the cell; f, cell fragment; g, cell aggregates. (**G**–**I**) Transmission electron microscopy photos of *C. pyrenoidosa* at concentrations of 0, 0.5, and 1.0 mg/L Na_2_SeO_4_, respectively (8000 times magnification). (**J**–**L**) Ultrastructure of *C. pyrenoidosa* at 0, 0.5, and 1.0 mg/L Na_2_SeO_4_ concentrations, respectively (15,000 times magnification). p, pyrenoids; st, starch plate; thy, thylakoids; ch, chloroplast; n, nucleus; li, lipid droplet; cw, cell wall; and cm, cell membrane.

As shown in Figure 3A–C, the microscopic observation results showed that under no Na_2_SeO_4_ treatment, the number of *C. pyrenoidosa* with the diameter range of 1–3 μm and 3–5 μm was 47 and 11, respectively (Table 1); under the 0.5 mg/L Na_2_SeO_4_ treatment, the number of *C. pyrenoidosa* with diameter ranges of 1–3 μm and 3–5 μm was 12 and 37, respectively (Table 1). Under the 1.0 mg/L Na_2_SeO_4_ treatment, the number of *C. pyrenoidosa* with diameter ranges of 1–3 μm and 3–5 μm was 22 and 11, respectively (Table 1), accompanied by *C. pyrenoidosa* cell splitting (a), cell agglomeration (b), and cavity phenomena (c) of *C. pyrenoidosa*. SEM observation showed that the surface of *C. pyrenoidosa* under 0 or 0.5 mg/L Na_2_SeO_4_ treatments was smooth, exhibiting obvious clear ridges, and the space interval between *C. pyrenoidosa* was appropriate without the particle structure (Figure 3D–F). Under the 1 mg/L Na_2_SeO_4_ treatment, the surface of the *C. pyrenoidosa* became rough; the ridges on the surface were relatively blurred; the *C. pyrenoidosa* membrane began to indent inward; the cell morphology became distorted; and fragmented structures and agglomeration phenomenon appeared between *C. pyrenoidosa* (Figure 3F). As shown in Figure 3G–L, TEM observation showed that under the 0 or 0.5 mg/L Na_2_SeO_4_ treatment, the *C. pyrenoidosa* cell wall (cw) and cell membrane (cm) edges were relatively neat, smooth, and closely connected; chloroplasts (ch) occupied almost the entire cell, and leaf-shaped chloroplasts were close to the periphery of the *C. pyrenoidosa*; thylakoids (th), taking on lamellar layers, were distributed in the chloroplasts; in the cytoplasm, the pyrenoids (p) were clearly visible, which were surrounded by a layer of evenly distributed starch granules. As shown in Figure 3L, when *C. pyrenoidosa* were treated with a concentration of 1 mg/L Na_2_SeO_4_, the *C. pyrenoidosa* membrane gradually became rough, indented, or even disrupted; the cytoplasm–cell wall separation phenomenon gradually became serious; the starch (st) granules gradually became larger, thus gradually crowding out thylakoids and occupying their spatial position in chloroplasts; the lamellar structure of thylakoids became too fuzzy to be distinguished, and the shape was distorted; the pyrenoid became slightly larger; and the volume of lipid (li) droplets became larger.

### 3.4. Nutritional Components of C. pyrenoidosa

**Figure 4:**
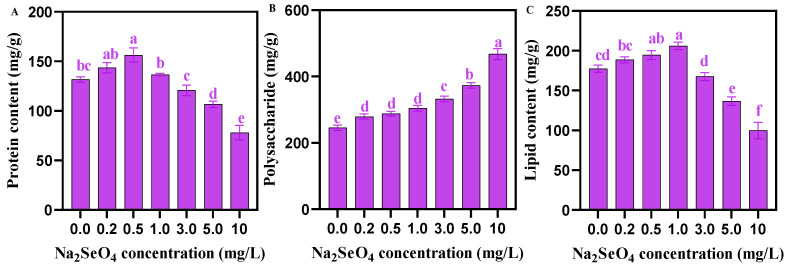
Nutrients in *C. pyrenoidosa* under different Na_2_SeO_4_ treatments. (**A**) Soluble protein content of *C. pyrenoidosa*. (**B**) Total polysaccharide content of *C. pyrenoidosa*. (**C**) Total lipid content of *C. pyrenoidosa*. Data are expressed as mean ± SD (standard deviation) (*n* = 3). Different lowercase letters represent significant differences. *p* ≤ 0.05 is considered statistically significant.

The soluble protein content in *C. pyrenoidosa* first showed a trend of increasing (0–0.5 mg/L) and then decreasing (0.5–10 mg/L), with the increasing concentration of applied Na_2_SeO_4_ (Figure 4A). The polysaccharide content in *C. pyrenoidosa* showed an increasing trend with the increase in the concentration of Na_2_SeO_4_ applied (Figure 4B). The lipid content in *C. pyrenoidosa* exhibited a trend of first increasing (0–1.0 mg/L) and then decreasing (1.0–10 mg/L), with the increasing concentration of Na_2_SeO_4_ (Figure 4C). Table 2 showed that the relative contents of total unsaturated fatty acids (TUFAs), total poly unsaturated fatty acids (TPUFA)s, n-3 series unsaturated fatty acids, n-6 unsaturated fatty acids, eicosapentaenoic acid (EPA), and docosahexaenoic acid (DHA), in *C. pyrenoidosa* first increased and then decreased as the concentration of Na_2_SeO_4_ increased, reaching the highest at a concentration of 0.5 mg/L Na_2_SeO_4_. The relative contents of total monounsaturated fatty acids (TMUFAs) and total saturated fatty acids (TSFAs) exhibited an opposite change trend, reaching the lowest value at the 0.5 mg/L Na_2_SeO_4_ concentration.

### 3.5. Analysis of Photosynthetic Pigment Content

**Figure 5:**
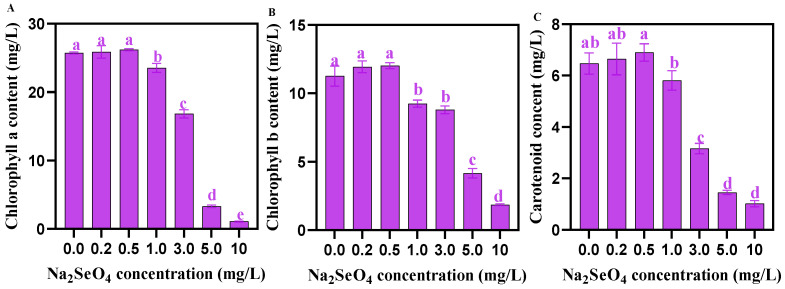
Chlorophyll contents in *C. pyrenoidosa* under different Na_2_SeO_4_ treatments. (**A**) Chla content in *C. pyrenoidosa.* (**B**) Chlb content in *C. pyrenoidosa*. (**C**) Carotenoid content in *C. pyrenoidosa.* Data are expressed as mean ± SD (standard deviation, *n* = 3). Different lowercase letters represent significant differences. *p* ≤ 0.05 is considered statistically significant.

The contents of Chla, Chlb, and carotenoids in *C. pyrenoidosa* showed no significant difference when the addition of Na_2_SeO_4_ under 0.5 mg/L. The addition of Na_2_SeO_4_ (1.0–10 mg/L) significantly reduced the contents of Chla, Chlb, and carotenoids in *C. pyrenoidosa* (Figure 5).

### 3.6. Activities of Antioxidant Enzymes in C. pyrenoidosa Under Different Concentrations of Na_2_SeO_4_ Treatments

**Figure 6:**
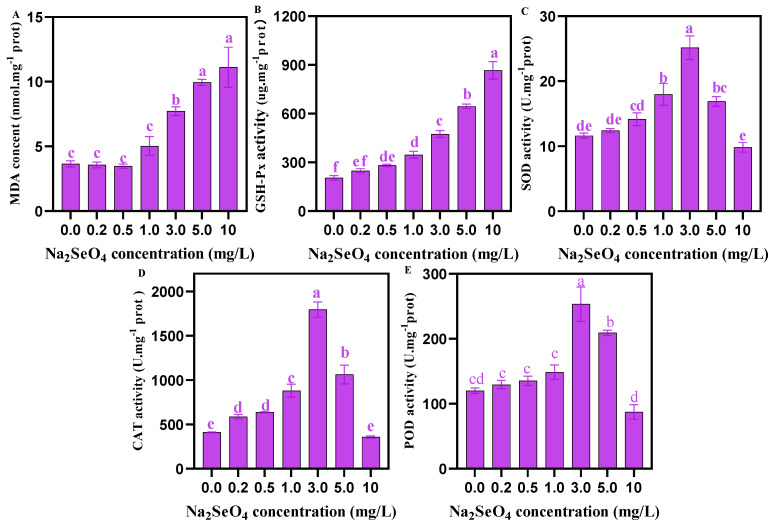
Activities of antioxidant enzymes in *C. pyrenoidosa* under different concentrations of Na_2_SeO_4_ treatments. (**A**) MDA content in *C. pyrenoidosa*. (**B**) GSH-Px activity in *C. pyrenoidosa*. (**C**) SOD activity in *C. pyrenoidosa*. (**D**) CAT activity in *C. pyrenoidosa*. (**E**) POD activity in *C. pyrenoidosa*. Data are expressed as mean ± SD (standard deviation) (*n* = 3). Different lowercase letters represent significant differences. *p* ≤ 0.05 is considered statistically significant.

As shown in Figure 6, at the Na_2_SeO_4_ addition concentration of 0–1.0 mg/L, the content of MDA did not change significantly, but when Na_2_SeO_4_ concentration was ≥1 mg/L, the content of MDA increased with the increase in Na_2_SeO_4_ concentration. The activity of GSH-Px in *C. pyrenoidosa* increased with the increase in Na_2_SeO_4_ concentration, but the activities of SOD, CAT, and POD first increased (0–3.0 mg/L Na_2_SeO_4_) and then decreased (3.0–10 mg/L Na_2_SeO_4_).

## 4. Discussion

Se has the dual functions of promoting growth and inhibiting toxicity of algae. In this study, we found that 0–0.5 mg/L Na_2_SeO_4_ promoted the growth of *C. pyrenoidosa*, while 1–10 mg/L Na_2_SeO_4_ inhibited its growth. Our microscopic and electron microscopic observations found that the addition of 0.5 mg/L Na_2_SeO_4_ resulted in plump *C. pyrenoidosa*. For example, the number of *C. pyrenoidosa* with the diameter range of 3–5 μm were 11, 37, and 11 under 0, 0.5, and 1.0 mg/L Na_2_SeO_4_ treatments, respectively (Figure 3A–C and Table 1). However, when the Na_2_SeO_4_ addition concentration was ≥1 mg/L, splitting, agglomeration, and cavity phenomena of *C. pyrenoidosa* were observed; the *C. pyrenoidosa* cell surface became rough; the *C. pyrenoidosa* membrane began to indent; and the thylakoid lamellar structure became blurred, which was accompanied by some other symptoms. The reason might be that the *C. pyrenoidosa* relieve Se toxicity and secrete some reducing substances that react with the Na_2_SeO_4_ on the *C. pyrenoidosa* cell surface, making *C. pyrenoidosa* surface rough, thus enhancing the light-shading effect on the algae body, ultimately affecting the structures of chloroplasts and the thylakoids [28]. The stable MDA level and the increased enzyme activities of GSH-Px, SOD, and CAT of *C. pyrenoidosa* under 0.5 mg/L Na_2_SeO_4_ treatment in the present study also supported the above results. In addition, when Na_2_SeO_4_ was ≥10 mg/L, red elemental Se appeared in the algae solution, which might be due to the reduction in oxidized Se to monomeric Se by *C. pyrenoidosa*, thus alleviating the oxidative damage caused by high Na_2_SeO_4_ concentration to the *C. pyrenoidosa* [29].

Although some studies on Se-rich *C. pyrenoidosa* have been conducted, most of them focus on total Se enrichment in *C. pyrenoidosa* [8,9,10], and report that the contents, Se-forms, and proportions of organic Se in *C. pyrenoidosa* are very limited. This study found that the total Se content of *C. pyrenoidosa* increased with the increase in the added Na_2_SeO_4_ concentration. However, as the concentration of Na_2_SeO_4_ increased, the proportion of organic Se in *C. pyrenoidosa* kept stable at (0–1.0 mg/L), and then decreased (3.0–10 mg/L); SeMet in organic Se accounted for the largest proportion; MeSeCys accounted for a smaller proportion; and SeCys_2_ was not detected. At the concentration of 0.5 mg/L Na_2_SeO_4_, the proportion of organic Se in *C. pyrenoidosa* was as high as 87.58%, and the content of SeMet was 61.84 μg/g. Studies reported that over 50% of dietary Se-supplementation for humans is in the form of SeMet [6]. In microalgae, SeMet, SeCys, and MeSeCys can be mutually converted through the Se metabolic pathway [19,30]. Usually, microalgae take up inorganic Se via sulfur-transport channels. Subsequently, they synthesize selenocysteine (SeCys) to fulfill their nutritional needs. Nevertheless, SeCys is further transformed into selenomethionine (SeMet). This process continues until methylation leads to the formation of volatile dimethyl selenide or dimethyl diselenide, which helps to reduce the accumulation of Se within the cells [31]. In this study, the content of SeMet was higher than that of SeCys_2_ and MeSeCys in the *C. pyrenoidosa*, which has also been reported in other studies by Mylenko et al. [7] and Guimarães et al. [32]. Similarly, Zhao et al. [9] found that the total Se content and organic Se content in *C. pyrenoidosa* increased with the increasingly applied Na_2_SeO_3_ concentration, but the organic Se conversion rate showed a gradual decreasing trend. Interestingly, under Na_2_SeO_3_ treatment, the organic Se form in *Tribonema minus* was mainly SeCys, followed by SeMet, which may be related to the mechanisms underlying different tolerances of different algae species to Se [19]. Our data also show that in the algae-water system, the proportion of Na_2_SeO_4_ in the remaining solution (after *C. pyrenoidosa*. extraction) showed a trend of first decreasing (0–0.5 mg/L) and then increasing (1.0–10 mg/L) as the Na_2_SeO_4_ concentration increased. Under the 0.5 mg/L Na_2_SeO_4_ treatment, the proportion of Se in the residual solution was reduced to 14.62%. These studies showed that the production of Se-rich *C. pyrenoidosa*, with a 0.5 mg/L concentration of Na_2_SeO_4_ can also reduce the Se residual solution pollution.

Proteins, lipids, and polysaccharides are the main organic nutrients in *C. pyrenoidosa*. This study found that compared with the control group, the protein and lipid contents in *C. pyrenoidosa* increased at a concentration of 1.0 mg/L Na_2_SeO_4_. This result was verified by our electron microscopic observation of the obvious increase in lipid droplets (Figure 3L). The possible reason for this result might be that the oxidative damage caused by Na_2_SeO_4_ needs to be repaired by antioxidant enzyme system, and thus, *C. pyrenoidosa*. have to synthesize more lipids to maintain the energy required for life activities [33]. Our data show that ≥ 3.0 mg/L Na_2_SeO_4_ concentration led to a significant decrease in protein and lipid contents, thus probably leading to the sub-health status or even death of *C. pyrenoidosa*. This result is consistent with previous research reports on other algae [19,20]. In contrast, accompanied by the significant decrease in protein and lipid contents, the polysaccharide content in *C. pyrenoidosa* continuously significantly increased with the increasing Na_2_SeO_4_ concentration, especially at the concentration of 3.0–10 mg/L Na_2_SeO_4_. The potential reason might be that the *C. pyrenoidosa* re-decomposed extra proteins other than those used for stress response and extra lipids, and other than those used for survival, reproduction, and membrane system construction into polysaccharides, so as to maintain the energy required for the life activities of the *C. pyrenoidosa* [34,35,36]. Previous research has indicated that *C. pyrenoidosa* can regulate the accumulation of its own proteins, polysaccharides, and lipids to adapt to different environments [37]. In addition, DHA- and EPA-rich algae oil is widely recognized as playing an important role in maintaining health and preventing cardiovascular and cerebrovascular diseases in organisms [38,39]. In this study, we observed the maximum relative contents of the TUFAs, essential fatty acids (EFA), n-3 series unsaturated fatty acids, n-6 series unsaturated fatty acids, unsaturated fatty acids, EPA, and DHA in *C. pyrenoidosa* cultured under the 0.5 mg/L Na_2_SeO_4_ treatment. Consistently, Pires et al. [16] have also found that the addition of appropriate amounts of Se can promote the synthesis of total unsaturated fatty acids in *C. pyrenoidosa*. In aquaculture, studies reported that *C. pyrenoidosa* are an excellent source of polyunsaturated fatty acids (PUFAs), particularly the n-3 series, which can replace fish oil and fish meal in diets [40,41].

Photosynthetic pigments are crucial for algae cells to absorb and convert light energy, and their contents are an important physiological indicator, reflecting the photosynthetic ability and the health status of algal cells [42]. In addition, photosynthetic pigments can also serve as a non-enzymatic antioxidant system to scavenge excited oxygen molecules and protect the photosynthetic membrane system [43]. In this study, we found that ≤0.5 mg/L exogenous Na_2_SeO_4_ concentration did not affect the synthesis of photosynthetic pigments in *C. pyrenoidosa*, while ≥1.0 mg/L Na_2_SeO_4_ concentration inhibited the synthesis of photosynthetic pigments in *C. pyrenoidosa*, and under ≥1.0 mg/L Na_2_SeO_4_ treatment, the changing trend of photosynthetic pigments in *C. pyrenoidosa* was highly consistent with that of *C. pyrenoidosa* biomass. These results are consistent with some previous reports [8,19]. Furthermore, our TEM results also showed that the chloroplast structure of *C. pyrenoidosa* was intact at ≤0.5 mg/L Na_2_SeO_4_ concentration, but it was destroyed at ≥1.0 mg/L Na_2_SeO_4_ concentration, further supporting that ≤0.5 mg/L exogenous Na_2_SeO_4_ was safe for *C. pyrenoidosa*.

MDA is an important parameter, reflecting the degree of peroxidative damage to *C. pyrenoidosa*. In this study, when the exogenous Na_2_SeO_4_ concentration was ≤0.5 mg/L, the content of MDA in *C. pyrenoidosa* did not change significantly. GSH-Px, SOD, CAT, and POD are the antioxidant enzymes which help to eliminate oxidative damage from the algae body. In this study, at ≤3.0 mg/L Na_2_SeO_4_ concentrations, the activities of GSH-Px, SOD, CAT, and POD in *C. pyrenoidosa* were increased with the increase in Na_2_SeO_4_ concentration, which might be because Se-stress will stimulate major enzymes represented by GSH-Px, SOD, CAT, and POD in the enzymatic antioxidant system to alleviate oxidative damage to *C. pyrenoidosa* caused by Na_2_SeO_4_. When Na_2_SeO_4_ was ≥5.0 mg/L, the activities of SOD, CAT, and POD enzymes in *C. pyrenoidosa* were decreased with the increasing concentration of Na_2_SeO_4_. The possible reason might be that excessive Na_2_SeO_4_ has exceeded the ultimate tolerance of the *C. pyrenoidosa*, thus resulting in the failure of the enzymatic antioxidant system. Our results are consistent with some previous reports [19,44,45]. Interestingly, when Na_2_SeO_4_ was ≥5.0 mg/L, GSH-Px enzyme activity increased with the increase in Na_2_SeO_4_ concentration, which might be explained by the higher Se tolerance of GSH-Px since Se is the enzyme active center of GSH-Px. Our results were supported by some previous findings that the higher Se stress intensity, the higher GSH-Px activity in *C. pyrenoidosa* [19].

## 5. Conclusions

In this study, for the first time, we explored the effects of Na_2_SeO_4_ on the *C. pyrenoidosa* growth, Se-form, and nutritional quality of *C. pyrenoidosa*. The results indicated that Na_2_SeO_4_ exerted a dose-dependent effect on the growth of *C. pyrenoidosa*. The growth of *C. pyrenoidosa* promoted under 0.5 mg/L Na_2_SeO_4,_ while it inhibited ≥1 mg/L Na_2_SeO_4_. At the concentration of 0.5 mg/L Na_2_SeO_4_, *C. pyrenoidosa* were plump and healthy; the content and proportion of organic-Se reached 64.20 μg/g and 87.58%; the main component of organic-Se is SeMet; the contents of biomass, soluble protein, lipids, and TPUFA reached the highest level. On the other hand, a 0.5 mg/L concentration of Na_2_SeO_4_ can also reduce the Se residual solution pollution. However, the addition of high concentration Na_2_SeO_4_ could inhibit the growth of *C. pyrenoidosa*, reduce the conversion rate of organic Se, and decrease the contents of protein and lipid. Overall, we recommended 0.5 mg/L Na_2_SeO_4_ for the production of Se-rich *C. pyrenoidosa*, which can not only produce high-quality *C. pyrenoidosa* but also environmental protection. Our findings will provide a theoretical basis for the development of algal health care products.

## Figures and Tables

**Table 1:** Diameter of *C. pyrenoidosa* under different Na_2_SeO_4_ concentrations.

Diameter (μm)	Na_2_SeO_4_ (mg/L)		
	0	0.5	1
	number		
1–2	3	0	1
2–3	44	12	21
3–4	10	30	9
4–5	1	7	2
Sum	58	49	33

**Table 2:** Fatty acid composition and relative content (%) in *C. pyrenoidosa* under different concentrtions of Na_2_SeO_4_ treatments.

Fatty Acid	Na_2_SeO_4_ (mg/L)			
	0	0.5	1	3
C14:0	0.31 ± 0.03 b	0.30 ± 0.03 b	0.33 ± 0.06 b	0.51 ± 0.09 a
C16:0	23.21 ± 1.83 a	23.14 ± 0.25 a	23.16 ± 1.25 a	20.98 ± 1.13 b
C17:0	0.21 ± 0.02 a	0.20 ± 0.04 a	0.20 ± 0.06 a	0.22 ± 0.02 a
C18:0	1.79 ± 0.36 ab	1.42 ± 0.18 b	2.50 ± 0.35 ab	4.58 ± 2.25 a
C22:0	0.37 ± 0.05 bc	0.20 ± 0.04 c	0.39 ± 0.12 b	0.61 ± 0.06 a
C24:0	0.21 ± 0.02 a	0.20 ± 0.05 a	0.26 ± 0.08 a	0.31 ± 0.09 a
C16:1	0.52 ± 0.03 a	0.61 ± 0.13 a	0.59 ± 0.06 a	0.51 ± 0.09 a
C18:1(n-9)	5.83 ± 0.84 a	4.68 ± 0.62 ab	3.69 ± 0.35 b	3.46 ± 0.54 b
C24:1	2.09 ± 0.10 a	2.39 ± 0.15 a	2.31 ± 0.11 a	2.04 ± 0.54 a
C22:1(n-9)	24.86 ± 0.11 c	20.17 ± 0.66 d	31.08 ± 2.28 b	39.6 ± 2.33 a
C18:3(n-3)	5.48 ± 0.57 b	6.58 ± 0.26 a	4.11 ± 0.35 c	2.54 ± 0.18 d
C20:3(n-3)	2.82 ± 0.10 b	3.05 ± 0.44 b	3.30 ± 0.35 b	4.48 ± 0.41 a
C20:5(n-3)	3.65 ± 0.11 b	4.68 ± 0.35 a	3.43 ± 0.35 b	3.57 ± 0.27 b
C22:6(n-3)	5.29 ± 0.16 b	6.81 ± 0.27 a	5.02 ± 0.46 b	5.40 ± 0.44 b
C18:2(n-6)	19.31 ± 1.38 a	20.64 ± 0.27 a	14.75 ± 0.11 b	5.70 ± 0.35 c
C18:3(n-6)	2.4 ± 0.36 a	3.15 ± 0.27 a	3.04 ± 0.26 a	3.06 ± 0.35 a
C20:4(n-6)	1.32 ± 0.03 b	1.51 ± 0.06 ab	1.45 ± 0.23 ab	1.83 ± 0.27 a
C22:2	0.32 ± 0.09 b	0.25 ± 0.04 b	0.38 ± 0.03 b	0.62 ± 0.04 a
the sum of the specific fatty acid subclass (%)				
TUFA	73.90 ± 2.52 a	74.53 ± 0.53 a	73.15 ± 2.73 a	72.8 ± 3.56 a
TSFA	26.10 ± 1.47 a	25.47 ± 0.36 a	26.85 ± 1.77 a	27.2 ± 3.53 a
TPUFA	40.59 ± 1.97 b	46.68 ± 0.32 a	35.48 ± 0.98 c	27.19 ± 1.08 d
TMUFA	33.31 ± 0.61 c	27.85 ± 0.37 d	37.67 ± 2.69 b	45.61 ± 2.49 a
n-3	17.25 ± 0.42 b	21.12 ± 0.36 a	15.86 ± 0.58 c	15.99 ± 0.44 bc
n-6	23.02 ± 1.73 b	25.31 ± 0.59 a	19.24 ± 0.46 c	10.58 ± 0.62 d

Notes: TFA, total fatty acids; TUFA, total unsaturated fatty acids; TSFA, total saturated fatty acids; TPUFA, total polyunsaturated fatty acids; TMUFA, total monounsaturated fatty acids; n-3, n-3 series unsaturated fatty acids; and n-6, n-6 series unsaturated fatty acids. Data are expressed as mean ± SD (standard deviation) (*n* = 3). Different lowercase letters in each line represent significant differences. *p* ≤ 0.05 is considered statistically significant.

## Data Availability

The original contributions presented in this study are included in the article. Further inquiries can be directed to the corresponding author.

## Abbreviations

CAT, catalase; Chla, chlorophyll a; Chlb, chlorophyll b; *C. pyrenoidosa*, *Chlorella pyrenoidosa*; GSH-Px, glutathione peroxidase; MDA, malonaldehyde; MeSeCys, methylselenocysteine; Na_2_SeO_4_, sodium selenate; Na_2_SeO_3_, sodium selenite; POD, peroxidase; PBS, phosphate buffer solution; Se, Selenium; SEM, scanning electron microscopy; SeCys_2_, selenocystine; SeCys, selenocysteine; SOD, superoxide dismutase; SeMet, selenomethionine; TEM, transmission electron microscopy.
